# Supplementary material for: Pseudomonas aeruginosa kills Staphylococcus aureus in a polyphosphate-dependent manner
Source: mSphere. 2024 Oct 4;9(10):e00686-24. doi: 10.1128/msphere.00686-24 (PMC11520310; doi:10.1128/msphere.00686-24)
Supplement: Supplemental figures — Figures S1 to S6. [file msphere.00686-24-s0001.pdf]

SUPPLEMENTARY INFORMATION

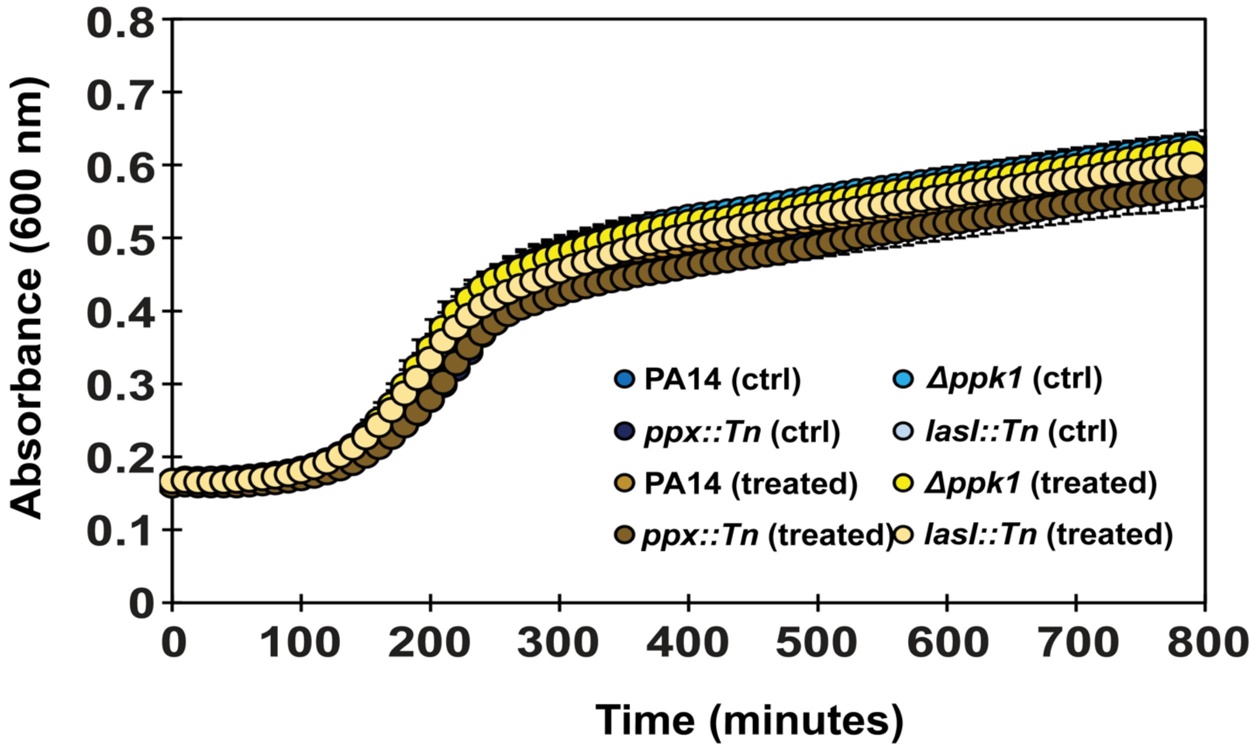

Supplementary FIG S1. *P. aeruginosa* is not inhibited by *S. aureus* spent media. The indicated *P. aeruginosa* strains were diluted into TSB to an OD_{600} of 0.03 and exposed to the spent media of a 24 hrs, stationary phase *S. aureus* culture in a 12.5:1 ratio. A_{600nm} was measured every 10 minutes for 16 hours using the Tecan Infinite 200 plate reader *(n = 3 [with 4 technical replicates], ± S.D.)*.

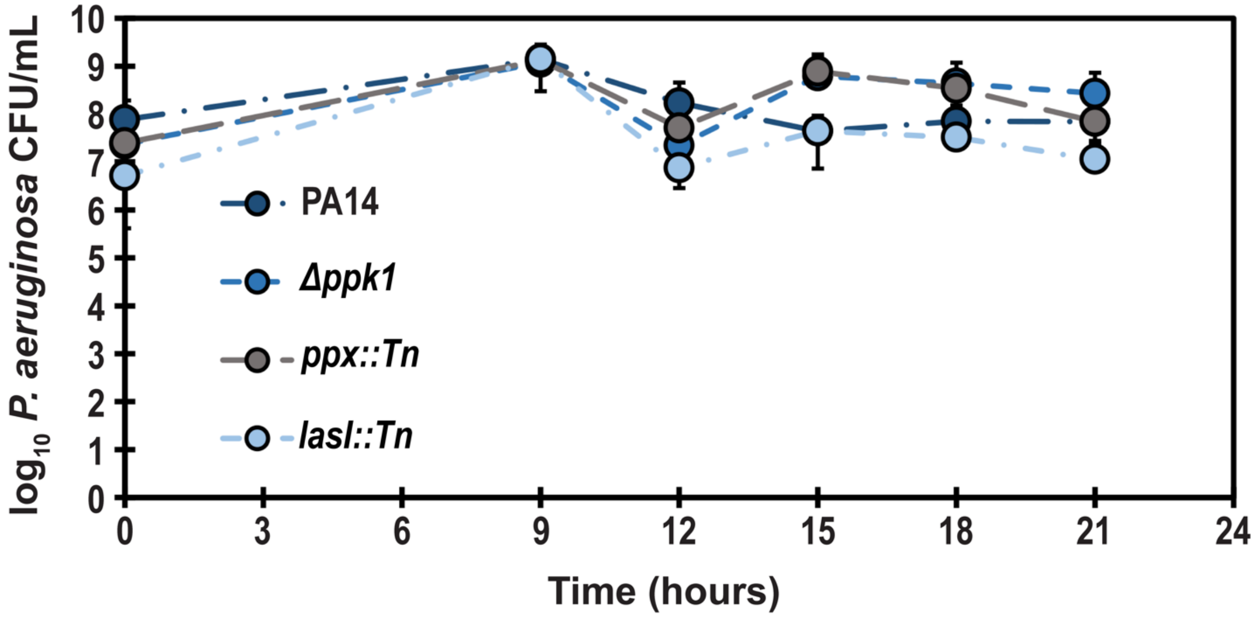

Supplementary FIG S2. *P. aeruginosa* survival is not affected by *S. aureus* in a co-cultivation assay. Overnight cultures of *S. aureus* strain USA300LAC and the indicated *P. aeruginosa* strains were each diluted into LB media to an OD_{600} of 1, mixed in a ratio of 100:1, and cultivated at 37 °C for 21 hrs. Samples were taken at indicated time points and serially diluted for *P. aeruginosa* CFU counts on cetrimide agar. Dark blue circles, PA14; blue circles, Δ*ppk*; bright blue circles, Δ*lasI;* grey circles, Δ*ppx; (n = 3, ± S.D.).*

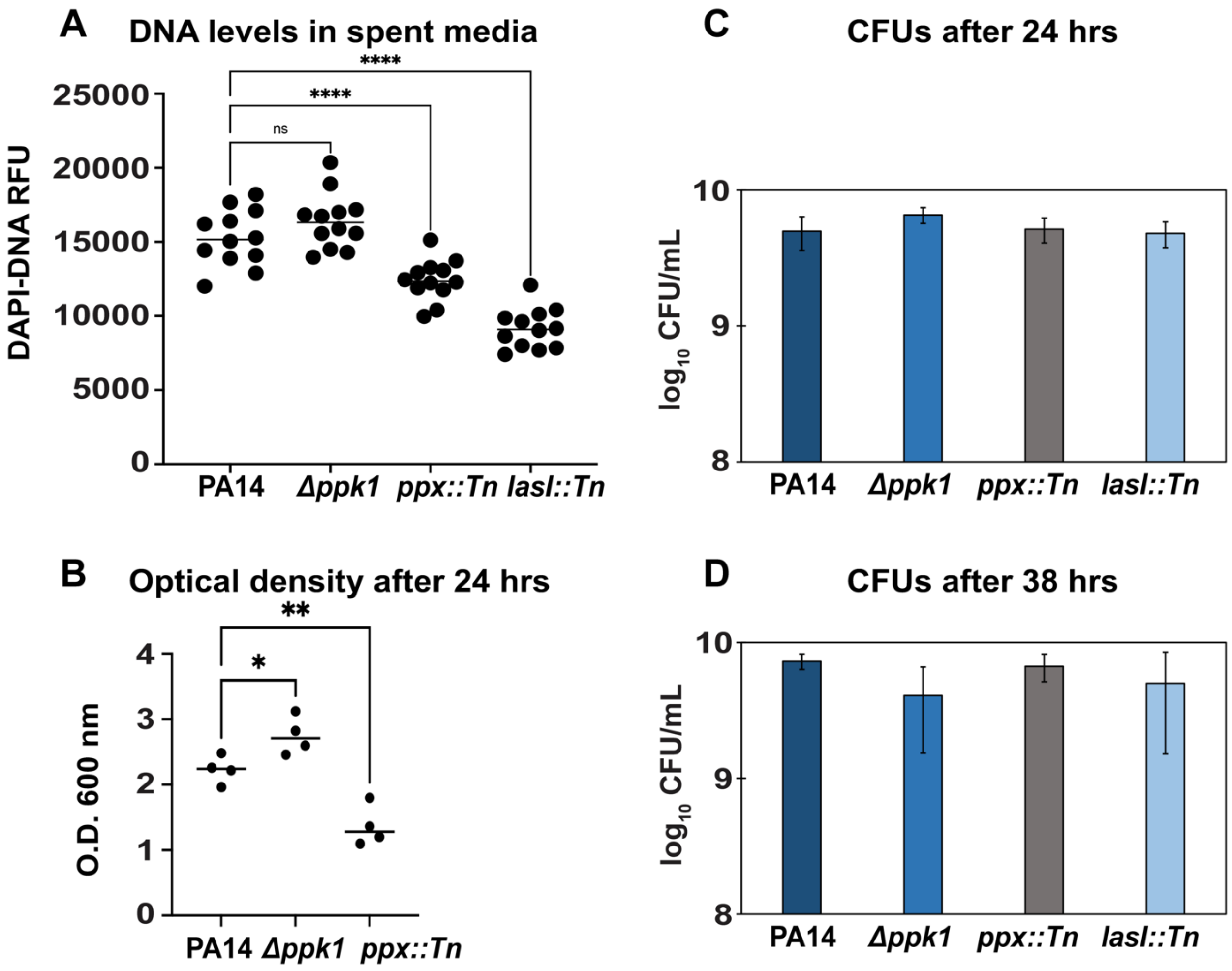

Supplementary FIG S3. ***P. aeruginosa*** **long-term survival is not affected by polyP production. (A)** Extracellular DNA levels were determined in the spent media of 24 hrs, stationary phase *P. aeruginosa* cultures using 25 μM DAPI. DAPI-DNA fluorescence was measured at excitation/emission wavelengths of 350 and 470 nm, respectively *(n = 8, ± S.D.).* **(B)** Optical densities of 24 hrs stationary phase PA14 strains were recorded; *(n = 4, ± S.D.)*. **(C & D)** Samples were taken from the indicated *P. aeruginosa* cultures after **(C)** 24 hrs and **(D)** 38 hrs of cultivation in LB at 37 °C and 300 rpm, serially diluted and plated onto LB agar for CFU counts; *(n = 3-4, ± S.D.)*. *Statistical tests: One-Way ANOVA, ns: p > 0.05, * p ≤ 0.05, ** p ≤ 0.01, **** p ≤ 0.0001).*

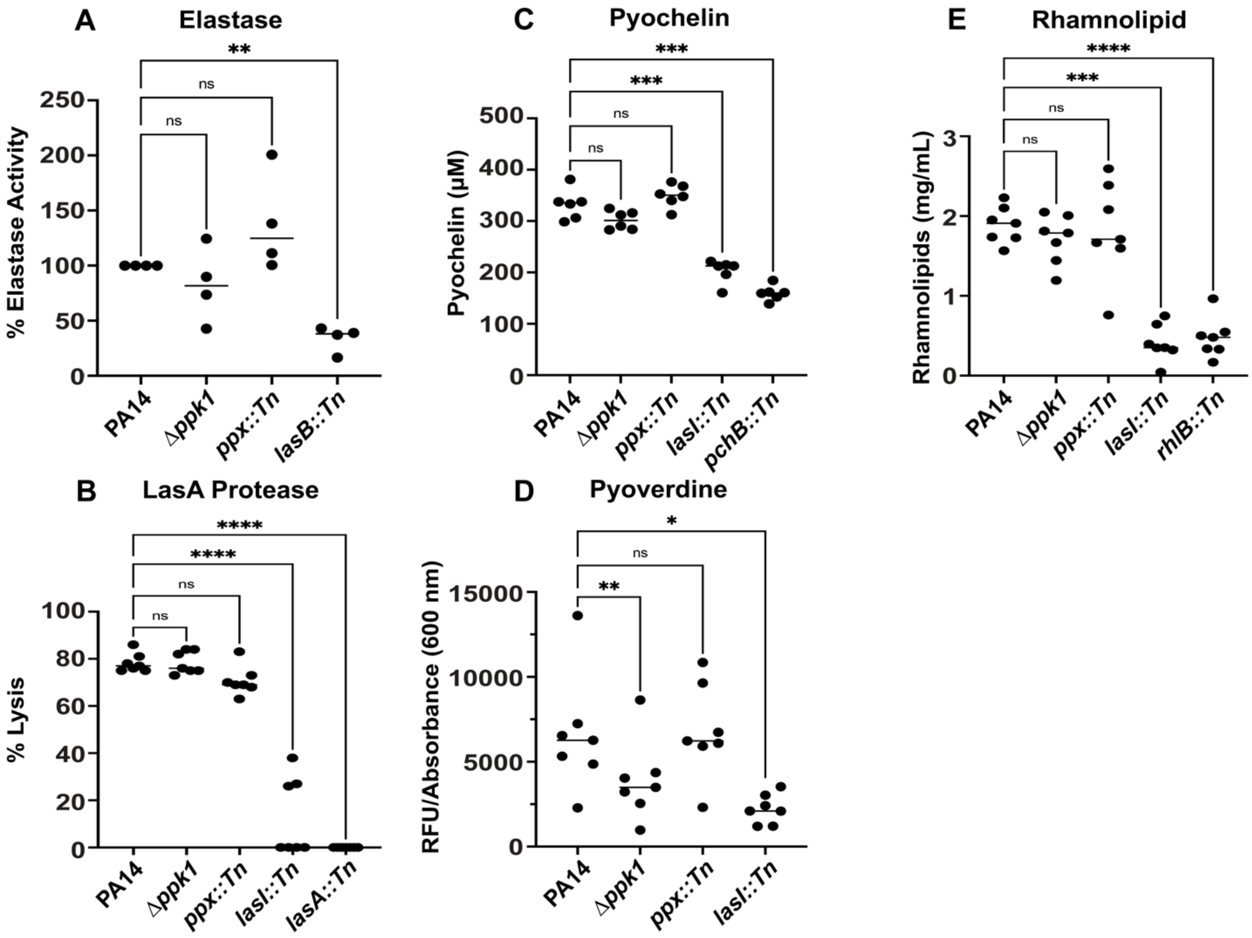

Supplementary FIG S4. The impact of polyP metabolism on *P. aeruginosa* virulence factors. The following virulence factors were quantified from sterile-filtered spent media of the indicated 24 hrs stationary phase *P. aeruginosa* cultures as described in *Material and Methods*: **(A)** Elastase *(n = 5, ± S.D.)*; **(B)** LasA Protease *(n = 7, ± S.D.)*; **(C)** Pyochelin *(n = 6, ± S.D.)*; **(D)** Pyoverdine *(n = 6, ± S.D.)*; and **(E)** Rhamnolipids *(n = 7, ± S.D.)*. *Statistical tests: One-Way ANOVA, ns: p > 0.05, * p ≤ 0.05, ** p ≤ 0.01, *** p ≤ 0.001, **** p ≤ 0.0001).*

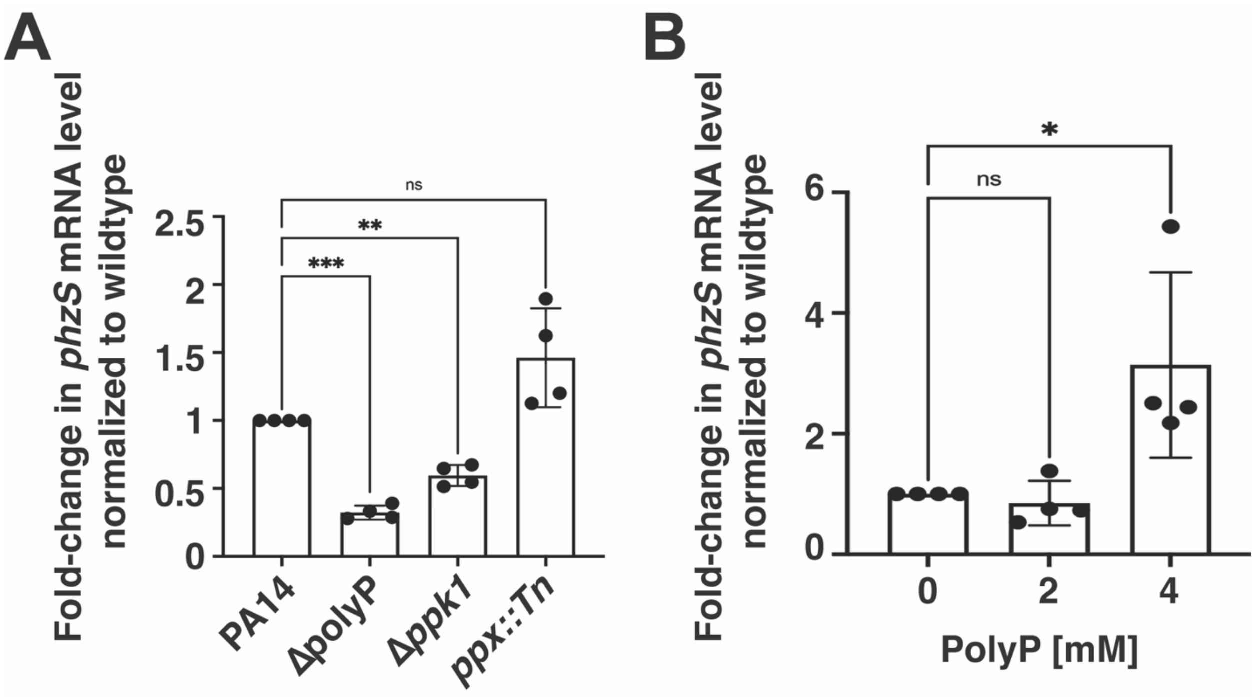

Supplementary FIG S5. The impact of polyP metabolism on transcription of *P. aeruginosa phzS*. (A) The indicated strains were cultivated in LB for 24 hrs under aerobic conditions. **(B)** PA14ΔpolyP was cultivated in in the presence and absence of the indicated polyP concentrations for 24 hrs under aerobic conditions. *phzS* transcript level were determined using qRT-PCR; *(n = 3, ± S.D.).* Statistical tests: *One-Way ANOVA, ns: p > 0.05, * p ≤ 0.05, ** p ≤ 0.01, *** p ≤ 0.001).*

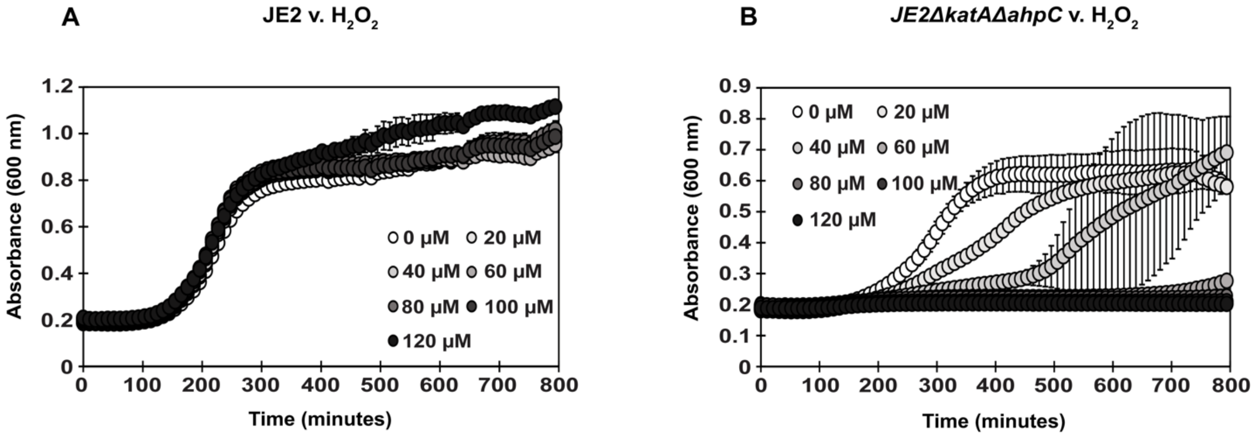

Supplementary FIG S6. Lag phase extension assays of different *S. aureus* strains. *S. aureus* strains reader **(A)** USA300LAC and **(B)** USA300LAC*ΔkatAΔahpC* were diluted into TSB to an OD_{600} of 0.03 and cultivated with and without the indicated concentrations of H_2O_2. A_{600nm} was measured every 10 minutes for 16 hours using the Tecan Infinite 200 plate reader, *(n = 3 [with 2 technical replicates], ± S.D.)*
